# The microRNA Expression in Crypt-Top and Crypt-Bottom Colonic Epithelial Cell Populations Demonstrates Cell-Type Specificity and Correlates with Endoscopic Activity in Ulcerative Colitis

**DOI:** 10.1093/ecco-jcc/jjae108

**Published:** 2024-07-18

**Authors:** Ruta Inciuraite, Rima Ramonaite, Juozas Kupcinskas, Indre Dalgediene, Ugne Kulokiene, Vytautas Kiudelis, Greta Varkalaite, Aurelija Zvirbliene, Laimas Virginijus Jonaitis, Gediminas Kiudelis, Andre Franke, Stefan Schreiber, Simonas Juzenas, Jurgita Skieceviciene

**Affiliations:** Institute for Digestive Research, Academy of Medicine, Lithuanian University of Health Sciences, Kaunas, Lithuania; Institute for Digestive Research, Academy of Medicine, Lithuanian University of Health Sciences, Kaunas, Lithuania; Institute for Digestive Research, Academy of Medicine, Lithuanian University of Health Sciences, Kaunas, Lithuania; Department of Gastroenterology, Academy of Medicine, Lithuanian University of Health Sciences, Kaunas, Lithuania; Institute of Biotechnology, Life Sciences Center, Vilnius University, Vilnius, Lithuania; Institute for Digestive Research, Academy of Medicine, Lithuanian University of Health Sciences, Kaunas, Lithuania; Institute for Digestive Research, Academy of Medicine, Lithuanian University of Health Sciences, Kaunas, Lithuania; Department of Gastroenterology, Academy of Medicine, Lithuanian University of Health Sciences, Kaunas, Lithuania; Institute for Digestive Research, Academy of Medicine, Lithuanian University of Health Sciences, Kaunas, Lithuania; Institute of Biotechnology, Life Sciences Center, Vilnius University, Vilnius, Lithuania; Institute for Digestive Research, Academy of Medicine, Lithuanian University of Health Sciences, Kaunas, Lithuania; Department of Gastroenterology, Academy of Medicine, Lithuanian University of Health Sciences, Kaunas, Lithuania; Institute for Digestive Research, Academy of Medicine, Lithuanian University of Health Sciences, Kaunas, Lithuania; Department of Gastroenterology, Academy of Medicine, Lithuanian University of Health Sciences, Kaunas, Lithuania; Institute of Clinical Molecular Biology, Christian-Albrechts-University of Kiel, Kiel, Germany; Institute of Clinical Molecular Biology, Christian-Albrechts-University of Kiel, Kiel, Germany; Institute for Digestive Research, Academy of Medicine, Lithuanian University of Health Sciences, Kaunas, Lithuania; Institute of Biotechnology, Life Sciences Center, Vilnius University, Vilnius, Lithuania; Institute for Digestive Research, Academy of Medicine, Lithuanian University of Health Sciences, Kaunas, Lithuania

**Keywords:** Colon crypt-bottom epithelial cells, colon crypt-top epithelial cells, microRNA, ulcerative colitis

## Abstract

**Background and Aims:**

Colonic epithelial barrier dysfunction is one of the early events in ulcerative colitis [UC], and microRNAs [miRNAs] participate in its regulation. However, the cell type-specific miRNome during UC remains unknown. Thus, we aimed to explore miRNA expression patterns in colon tissue and epithelial cells during active and quiescent UC.

**Methods:**

Small RNA-sequencing in colon tissue, crypt-bottom [CD44^+^], and crypt-top [CD66a^+^] colonic epithelial cells from two cohorts of UC patients [*n* = 74] and healthy individuals [*n* = 50] was performed. Data analysis encompassed differential expression, weighted gene co-expression network, correlation, and gene-set enrichment analyses.

**Results:**

Differentially expressed colonic tissue miRNAs showed potential involvement in the regulation of interleukin-4 [IL-4] and IL-13 signalling during UC. As this pathway plays a role in intestinal barrier regulation, consecutive analysis of spatially distinct colonic epithelial cell populations was performed. Cell-type- [crypt-top and crypt-bottom] specific miRNA expression patterns were identified in both active and quiescent UC. Target genes of differentially expressed epithelial miRNAs under different disease activity were overrepresented in epithelial cell migration and therefore intestinal barrier integrity regulation. The pro-inflammatory miRNA co-expression module M1 correlated with endoscopic disease activity and successfully distinguished active and quiescent UC not only in both epithelial cell populations, but also in the colon tissue. The anti-inflammatory module M2 was specific to crypt-bottom cells and was significantly enriched in quiescent UC patients.

**Conclusions:**

miRNA expression was specific to colonic epithelial cell populations and UC state, reflecting endoscopic disease activity. Irrespective of the UC state, deregulated epithelial miRNAs were associated with regulation of intestinal barrier integrity.

## 1. Introduction

Colonic epithelial cells and their secreted products primarily form and maintain frontline intestinal barrier function which deteriorates early in ulcerative colitis [UC].^1^ Colonic crypt epithelial cells such as colonocytes, goblet, and epithelial stem cells have been shown to play an important role in UC pathogenesis via reduced epithelial mucus secretion and/or increased barrier permeability.^2^ Since one of the aims in UC management is to induce and then to maintain remission, disentangling the molecular mechanisms regulating epithelial barrier may help to modulate its permeability and retain long-lasting remission in UC.

Recent studies provide evidence for microRNAs [miRNAs] as being involved in the regulation of intestinal epithelial integrity and barrier permeability via interference with protein-coding genes responsible for tight and adherent junctions,^3^ also in UC.^4^ However, most of the studies have been based on either immortalized cell cultures or bulk tissue experiments, and thus the exact cellular context of miRNA deregulation during colonic inflammation remains elusive. Therefore, the search for cell type-specific deregulation of miRNAs in UC might uncover novel aspects of underlying regulatory pathways and molecular targets for therapeutic development.

Here, we report the comprehensive miRNA expression profiling on colon tissue and colonic crypt-bottom [CD44^+^] and crypt-top [CD66a^+^] cell population levels in active and quiescent UC. By using small RNA-sequencing [RNA-seq] we determine UC activity-specific, as well as epithelial cell population-specific, miRNA expression signatures during colonic inflammation. Additionally, we describe the miRNA co-expression network and evaluate its relationships to clinical characteristics of UC. Finally, we explore putative biological pathways in which the deregulated miRNAs are involved and provide potential miRNA candidates for further development of UC management.

## 2. Materials and methods

### 2.1. Ethics and consent

The approvals to perform the study was received from Kaunas Regional Biomedical Research Ethics Committee [No. BE-2-31, 22-03-2018, No. BE-2-8, 19-02-2013]. All subjects signed a written informed consent form to participate in the study.

### 2.2. Study design

The study comprised two distinct yet interconnected parts—Study parts I and II. In Study part I, miRNA profiling was performed in colon tissue samples of Study group I. Then, *IL-4* and *IL-13* expression patterns were analysed in colon tissue samples of Study group III. In Study part II, fluorescence-activated cell sorting [FACS] was performed on colon tissue samples of Study group II to enrich two distinct epithelial cell populations, followed by miRNA expression analysis. Subsequently, miRNA co-expression modules were identified within the epithelial cell populations and finally validated in the colon tissue samples from the first part of the study. The flowchart illustrating the study design and methodology is presented in the Supplementary Figure S1.

### 2.3. Study samples

Study subject recruitment was conducted at the Department of Gastroenterology, Lithuanian University of Health Sciences [Kaunas, Lithuania] during the period 2011–2014 [Study groups I and III] and 2017–2019 [Study group II]. For miRNA sequencing, colon biopsies were collected from two independent study subject groups (Study group I [*n* = 76] and group II [*n* = 48]). Additionally, colon biopsies for quantitative (q)PCR-based gene expression analysis were collected in the third independent group (Study group III [*n* = 200]). Demographic and clinical characteristics of all study subjects are presented in Table 1. Colon tissue samples of patients with active UC and quiescent UC were collected during routine colonoscopy performed as a part of their planned examination programme. The diagnosis of UC was based on standard clinical, endoscopic, radiological, and histological criteria.^5^ Endoscopic activity was determined using the Mayo endoscopic subscore.^6^ Quiescent UC was confirmed in patients with stool frequency ≤3/day, no rectal bleeding, and healed mucosa at endoscopy [endoscopic Mayo subscore ≤1]. The control individuals included in all study groups consisted of healthy subjects who either underwent colonoscopy due to a positive faecal occult blood test or were consulted on functional complaints, but had a normal colonoscopy, uninflamed mucosa during histopathological examination, and no previous history of intestinal inflammation. All patients enrolled in the study were of European descent.

**Table 1.** Demographic and clinical characteristics of the subjects

	Study group I, *n* = 76			Study group II, *n* = 48			Study group III, *n* = 200		
	Active UC, *n* = 23	Quiescent UC, *n* = 20	HC, *n* = 33	Active UC, *n* = 16	Quiescent UC, *n* = 15	HC, *n* = 17	Active UC, *n* = 75	Quiescent UC, *n* = 50	HC, *n* = 75
Age, years									
Mean ± SD	43.9 ± 16.1^*^	47.2 ± 13.5 ^*^	58.5 ± 12.7 ^*^	41.6 ± 16.3 ^**^	57.3 ± 12.1 ^**^	50.5 ± 16.1	46.1 ± 13.7	47.3 ± 16.1	46.3 ± 11.9
Sex, *n* [%]									
Female	10 [43.5]	9 [45.0]	21 [73.6]	4 [25.0] ^***^	10 [66.7] ^***^	10 [58.8] ^***^	37 [49.3]	24 [48.0]	38 [50.7]
Full Mayo score, *n* [%]									
Remission [0–2]	0 [0.0]	20 [100.0]	–	0 [0.0]	15 [100.0]	–	0 [0.0]	50 [100.0]	–
Mild [3–5]	1 [4.4]	0 [0.0]	–	4 [25.0]	0 [0.0]	–	3 [4.0]	0 [0.0]	–
Moderate [6–10]	21 [91.3]	0 [0.0]	–	11 [68.8]	0 [0.0]	–	70 [93.3]	0 [0.0]	–
Severe [10–12]	1 [4.4]	0 [0.0]	–	1 [6.3]	0 [0.0]	–	2 [2.7]	0 [0.0]	–
Endoscopic Mayo subscore, *n* [%]									
Normal to mild [0–1]	0 [0.00]	20 [100.0]	33 [100.0]	0 [0.0]	15 [100.0]	17 [100.0]	0 [0.0]	50 [100.0]	75 [100.0]
Moderate to severe [2–3]	23 [100.0]	0 [0.0]	–	16 [100.0]	0 [0.0]	–	75 [100.0]	0 [0.0]	–
Smoking status, *n* [%]									
Never	8 [34.8]	11 [55.0]	23 [69.7]	9 [56.3]	10 [66.7]	12 [70.6]	43 [57.3]	24 [48.0]	43 [57.3]
Smoking	8 [34.8]	6 [30.0]	5 [15.2]	3 [18.8]	1 [6.7]	3 [17.7]	8 [10.7]	8 [16.0]	17 [22.7]
Ex-smoker	3 [13.1]	2 [10.0]	1 [5.0]	4 [25.0]	4 [26.7]	2 [11.8]	22 [29.3]	16 [32.0]	12 [16.0]
Unknown	4 [17.4]	1 [5.0]	1 [3.0]	–	–	–	2 [2.7]	2 [4.0]	3 [4.0]
BMI, kg/m^2^									
Median, [range]	26.8 [18.1–56.2]	26.2 [19.7–31.5]	27.7 [19.6–39.5]	25.5 [18.0–37.2]	28.4 [19.8–32.7]	25.9 [18.9–31.9]	24.9 [16.2–41.5]	25.8 [19.5–34.6]	24.9 [16.9–49.3]
Unknown, *n* [%]	1 [4.4]	1 [5.0]	2 [6.1]	–	1 [6.7]	–		2 [4.0]	5 [6.7]

^*^Age differed between both active and quiescent UC groups, and HC, estimated *p* value <0.05 [*t*-test].

^**^Age differed between both active and quiescent UC groups, and HC, estimated *p* value <0.05 [*t*-test].

^***^Sex distribution differed between three groups, estimated *p* value <0.05 [Pearson’s chi-squared test].

### 2.4. Colon tissue disaggregation

Colon biopsies were mechanically and enzymatically separated into single-cell suspensions. Briefly, four to six biopsies [5–10 mg wet weight each] were washed with PBS solution containing 50 IU/mL penicillin, 50 µg/mL streptomycin, and 0.5 mg/mL gentamicin. Biopsies were minced into small pieces [~1–2 mm^3^] and incubated in 1× trypsin-EDTA solution for 40–45 min at room temperature on an agitator to dissociate single epithelial cells from the lamina propria. After incubation, tissue fragments were gently transferred into PBS and shaken. The isolated cell suspension was filtered and suspended in DMEM/Ham’s F-12 medium [1:1] with 15 mM HEPES [Gibco] buffer for flow cytometry procedures.

### 2.5. Flow cytometry and FACS

To minimize the loss of cell viability, fresh cell suspensions prepared shortly before flow cytometry were used in all cell sorting experiments. Antibody staining was performed in PBS supplemented with 1% heat-inactivated fetal bovine serum. To minimize non-specific binding of antibodies, cells were first incubated with Human TruStain FcX [Fc Receptor Blocking Solution; BioLegend] for 10 min at a concentration of 3–10 × 10^5^ cells/100 µL. Cells were subsequently stained without washing with antibodies at dilutions recommended by the manufacturers. Antibodies used in this study were selected based on previous study^7^ and included: mouse anti-human CD326/EpCAM-FITC [clone VU-1D9, Life Technologies], mouse anti-human CD44-APC [clone G44-26, BD Biosciences], and mouse anti-human CD66a-PE [clone 283340, R&D Systems]. Cells positive for expression of non-epithelial lineage markers were excluded by staining with mouse anti-human CD45-APC-Cy7 [clone 2D1, BioLegend] [see gating strategy in Supplementary Figure S2A]. After 20 min, stained cells were washed of excess unbound antibodies and resuspended in PBS supplemented with 1% heat-inactivated fetal bovine serum. Flow-cytometry analysis was performed using a CyFlow Space cell sorter [Sysmex Partec] [see representative plots of flow cytometry data in Supplementary Figure S2B–D]. In cell sorting experiments, each population of interest was sorted individually, using a protocol already built-in within the CyFlow Space flow cytometer software package [FloMax 2.8], with appropriate adjustments. Data were analysed and visualized using FlowJo v10.7 [BD FlowJo]. Two cell populations [CD45^−^/EpCAM^+^/CD44^+^/CD66a^+^ and CD45^−^/EpCAM^+^/CD44^−^/CD66a^+^] were further described based on the literature and a single-cell RNA-seq dataset of the human colon, available in the GEO database with accession number GSE116222 [see the single-cell RNA-seq data-derived expression plot of three main cell surface markers in Supplementary Figure S3]. The CD45^−^/EpCAM^+^/CD44^+^/CD66a^−^ population was entitled as crypt-bottom cells [consisting of undifferentiated colonic epithelial cells as well as secretory goblet and enteroendocrine cells], while the CD45^−^/EpCAM^+^/CD44^−^/CD66a^+^ population was entitled as crypt-top cells [consisting of absorptive colonocytes and BEST4^+^/OTOP2^+^ cells]. Sorted cell samples were frozen and stored at −70°C prior to total RNA extraction.

### 2.6. Total RNA extraction

Total RNA extraction from colon biopsies and sorted cells was performed using standard protocols of a commercial miRNeasy Mini Kit [Qiagen] and Single Cell RNA Purification Kit [Norgen, Canada], respectively. Total RNA concentration was evaluated via a NanoDrop2000 spectrophotometer [Thermo Scientific] and Qubit 4 fluorometer [Invitrogen]. Total RNA quality was assessed using an Agilent 2100 Bioanalyzer [Agilent Biotechnologies].

### 2.7. Quantitative reverse transcription PCR [RT-qPCR] and data analysis

To estimate expression of the *IL-4* and *IL-13* genes in colon tissue of UC patients, total RNA from colon tissue samples was reverse transcribed using a High-Capacity cDNA Reverse Transcription Kit [Applied Biosystems]. Further, expression levels were measured using TaqMan Gene Expression Assays [Assay IDs: *IL-4* Hs00174122_m1; *IL-13* Hs00174379_m1] on the 7500 Fast Real-Time PCR System [Applied Biosystems]. The cycle threshold [*C*_T_] values of *IL-4* and *IL-13* were normalized to the value of the *GAPDH* [Assay ID: Hs99999905_m1] reference gene. All procedures were performed in accordance with the manufacturer’s protocol. Statistical analysis was performed using R studio software [v.4.0.3]. Data distribution was determined using the Shapiro–Wilk test, and gene expression differences were analysed using the two-sided Mann-Whitney U test. Differences between the values were considered significant at *p* < 0.05.

### 2.8. Preparation of small RNA libraries and next-generation sequencing

Small RNA libraries from tissue samples were prepared using the TruSeq Small RNA Sample Preparation Kit [Illumina] with 1 µg of total RNA input per sample. Small RNA libraries from sorted cells were prepared using the NEXTFLEX Small RNA-seq Kit v.3 [Bioo Scientific] with up to 50 ng of total RNA input per sample. Procedures were conducted according to the manufacturers’ protocols. The yield of sequencing libraries was assessed using the Agilent 2200 TapeStation system [Agilent Biotechnologies]. Subsequently, the TruSeq libraries were pooled with around 24 samples per lane, while the NEXTFLEX libraries were pooled with around 16 samples per lane and then sequenced on a HiSeq 4000 [Illumina] next-generation sequencing platform.

### 2.9. Bioinformatics and statistical analysis of small RNA-seq data

The demultiplexed raw reads [.fastq] were processed with the nf-core/smrnaseq v.1.0.0 best-practice analysis pipeline using default parameters [Nextflow v.20.01.0].^8^ The pipeline was executed within a Docker container. Briefly, depending on the small RNA-seq library preparation kits, the ‘illumina’ or ‘nextflex’ protocol was selected for processing of the libraries generated from tissue and sorted cell samples, respectively. First, Trim Galore [v.0.6.3] was used to remove 3ʹ adapter [5ʹ-TGGAATTCTCGGGTGCCAAGG-3ʹ for both ‘illumina’ and ‘nextflex’ protocols] sequences from the reads and an additional four nucleotides from both the 5ʹ and 3ʹ ends of reads [only for the ‘nextflex’ protocol]. Second, to reduce computational time, the reads with identical sequences were collapsed using seqcluster^9^ while saving read count information. Third, Bowtie1 v.1.2.2^10^ was used to perform the alignment of collapsed reads against mature and hairpin miRNA sequences in miRbase database v.22.1.^11^ Finally, miRNA annotation was performed using mirTOP v.0.4.23.^12^ Further, sample and miRNA quality control [QC] was performed: samples with initial read count <1.5 interquartile range [IQR] and number of detected miRNAs < .5 IQR on log_2_ scale as well as non-expressed [mean raw count <1] and non-variable miRNAs were excluded from further analysis. Subsequently, to perform differential expression analyses of the size factor-normalized counts of mature miRNAs between samples, negative binomial generalized linear models implemented in the R package DESeq2^13^ were used including age [scaled and centred] and sex as covariates in the model. The *p*-values resulting from Wald tests were corrected for false discovery rate [FDR] according to Benjamini and Hochberg. miRNAs with FDR < 0.05 and absolute value of log_2_FC > 1 were considered to be significantly differentially expressed. A multidimensional scaling [MDS] analysis using Euclidean distance was performed on variance stabilizing transformation [VST] normalized miRNA count data. Additionally, Spearman’s rank correlation analysis was performed between sex- and age-adjusted normalized miRNA read counts and endoscopic Mayo subscore. FDR < 0.05 was considered statistically significant. Removal of sex and age effects from normalized data was performed using the removeBatchEffect function from the limma R package.^14^ Statistical analyses and data processing were performed using R v.4.0.3.^15^ Visualization of graphs was performed using the ggplot2 package.^16^

### 2.10. Gene set enrichment analysis

To obtain putative biological functions of differentially expressed miRNAs, gene set enrichment analysis [GSEA] was performed using Reactome pathways^17^ and Gene Ontology [GO] categories.^18^ More specifically, luciferase assay-validated miRNA-target interactions [MTIs] were obtained from miRecords,^19^ miRTarBase,^20^ and TarBase^21^ using the multiMiR package.^22^ The retrieved MTIs were then submitted to a hypergeometric test implemented in the enrichPathway [from the ReactomePA package^23]^ and enrichGO [from the clusterProfiler package^24]^ functions using genes that are expressed in colon crypt-bottom [CD44^+^] and crypt-top [CD66a^+^] cells as a background reference [defined as universe]. The universe genes were obtained from single-cell RNA-seq data of the human colon, available in the GEO database with accession number GSE116222.^25^ Pathways with FDR < 0.05 were considered to be significantly deregulated. The expression of IL-4 and IL-13 signalling pathway-related genes in different human colonic cell populations was also analysed using the GSE116222 dataset.

### 2.11. miRNA co-expression analysis

Samples of study group II were used for weighted gene co-expression network analysis [WGCNA] aiming to identify modules of co-expressed miRNAs using the CEMiTool package^26^ [v.1.22.0] for R. A VST normalized miRNA count table was used to generate the co-expression modules. Filtering based on variance was applied on the gene expression table prior to identification of miRNA co-expression modules. The minimal number of miRNAs per submodule as well as minimum size of gene sets for GSEA was set to five, while the *p*-value threshold for filtering was set to 0.1. Further, the eigengene value of the co-expression module identified in colonic epithelial cells was also calculated in colon tissue data using the expression table of co-expressed miRNAs and WGCNA package^27^ [v.1.72-1] for R. Next, Spearman’s rank correlation coefficients were calculated between module eigengene values and endoscopic Mayo subscore in both colonic epithelial cell populations as well as in colon tissue. FDR < 0.05 was considered statistically significant. Finally, to assess the performance of the identified module eigengene value for distinguishing between active and quiescent UC in both colon tissue and distinct colonic epithelial cell populations, analysis of area under the receiver operating characteristic curve [AUC-ROC] was applied using the pROC package^28^ [v.1.18.4] for R.

## 3. Results

### 3.1. Differentially expressed miRNAs in active and quiescent UC tissues are involved in regulation of inflammation-related pathways

**Figure 1.**
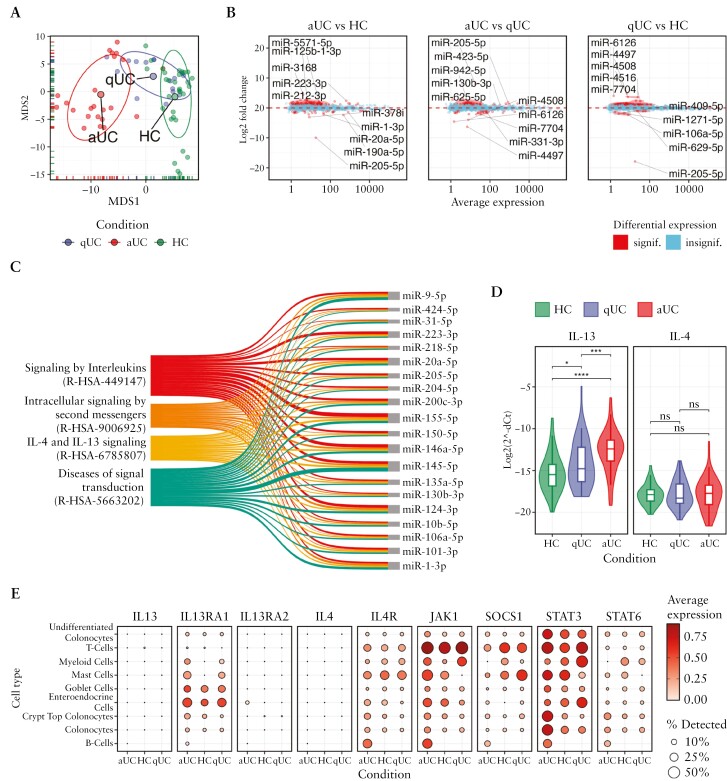
Small RNA-seq defines differentially expressed miRNAs involved in inflammation-associated pathways in active and quiescent UC tissues. [A] MDS plot showing the similarity structure of the miRNA transcriptomes in active UC [aUC] [*n* = 23], quiescent UC [qUC] [*n* = 20], and HC [*n* = 30] tissue samples based on normalized expression values. The dots represent samples coloured by group. The centroid of ellipses corresponds to the group mean; the shapes are defined by covariance within a given group. [B] Differentially expressed miRNAs in aUC [*n* = 23] and qUC [*n* = 20]. The red colour represents significantly [FDR < 0.05] differentially expressed miRNAs with an absolute value of log_2_FC > 1, while the blue colour represents non-differentially expressed miRNAs. [C] Sankey plot showing the overlapping significantly enriched [FDR < 0.05] Reactome pathways among the pairwise comparisons of aUC and qUC with HC tissues identified by miRNA set enrichment analysis [left side], and highlighting the top 20 miRNAs with the highest count of target genes within these pathways [right side]. Line width reflects the number of miRNA–target gene counts in the pathways. Line colour represents a distinct Reactome pathway. [D] Violin plots showing expression levels of *IL-4* and *IL-13* genes measured by RT-qPCR in colon tissue samples of an independent cohort of HC [*n* = 75], aUC [*n* = 75], and qUC [*n* = 50] individuals. Violin colour represents study group. Median of each group is represented by a vertical line. Quartiles of each group are represented by whiskers. Gene expression is represented on a logarithmic scale, and ΔCt values are inversed in order to show the true direction of the expression. Significance levels: *p ≤ 0.05, ***p ≤ 0.001, ****p ≤ 0.0001, ns—not significant. [E] Dot plot showing the expression of IL-4 and IL-13 signalling pathway-related genes in distinct epithelial and immune cell populations of human colon [dataset GEO accession number: GSE116222] within the aUC, qUC, and HC groups. The size of the dot represents the fraction of cells expressing a particular gene in each group [%]. The colour of the dot represents average expression of the gene.

To identify differentially expressed miRNAs and their putative regulatory processes during chronic colon inflammation, small RNA-seq was performed on inflamed [active] and non-inflamed [quiescent] colonic mucosal biopsies of UC patients and healthy controls [HC] [Figure 1].

After count data normalization and QC, 573 unique miRNAs were found to be expressed in colon tissue samples. The overall similarity structure [based on MDS analysis; see Methods] of colon miRNA transcriptomes revealed two clearly resolved clusters corresponding to active UC and HC tissues, while the third cluster corresponding to quiescent UC overlapped with both active UC and HC clusters, suggesting a shift of miRNA expression from a healthy to inflammatory state [Figure 1A]. To evaluate expression of specific miRNAs in active and quiescent UC, differential gene expression analysis was performed. As expected, the most profound miRNA deregulation was observed comparing active UC to HC or to quiescent UC (93 and 59 differentially expressed miRNAs [FDR < 0.05 and |log_2_FC| > 1], respectively). Interestingly, although substantially lower than in active UC, a differential expression of miRNAs [*n* = 32] was also observed in quiescent UC compared to HC [Figure 1B; Supplementary Table S1]. Among the differentially expressed miRNAs, a considerable number [*n* = 13] of molecules were deregulated in both active and quiescent UC compared to HC. In addition, the gradual decrease in miR-1-3p expression was found in all pairwise comparisons [Supplementary Table S1].

To further determine the biological function of differentially expressed miRNAs in the pathogenesis of UC, GSEA was performed for each pairwise comparison using validated target genes of significantly deregulated miRNAs and Reactome pathways [Supplementary Figure S4 and Table S2]. Intriguingly, both active UC and quiescent UC, compared to HC, had overrepresented interleukin signalling-related pathways among the top significant ones, such as ‘Signaling by Interleukins’ [R-HSA-449147], ‘Interleukin-4 and Interleukin-13 signaling’ [R-HSA-6785807], ‘Intracellular signaling by second messengers’ [R-HSA-9006925], and ‘Diseases of signal transduction by growth factor receptors and second messengers’ [R-HSA-5663202]. Based on the highest number of target genes, we identified 20 major miRNAs, including miR-223-3p, miR-20a-5p, miR-155-5p, miR-146a-5p, miR-1-3p, miR-31-5p, miR-10b-5p, and miR-205-5p, that were involved in these pathways [Figure 1C].

To explore the genes related to the interleukin-4 [IL-4] and IL-13 signalling pathway in colonic tissue more deeply, the expression patterns of two main cytokines, IL-4 and IL-13, were analysed using targeted RT-qPCR. A gradual increase in *IL-13* expression was observed between UC patients and HC individuals (2.19-fold [*p* = 0.031] increase in quiescent UC vs. HC, 2.91-fold [*p* = 0.0007] increase in active UC vs. quiescent UC, 6.38-fold [*p* = 2 × 10^−10^] increase in active UC vs. HC), whereas the expression of *IL-4* did not differ between the groups [Figure 1D]. Subsequently, the GSE116222 dataset was used to explore the expression signatures of IL-4 and IL-13 signalling-related genes, also identified as validated target genes of differentially expressed colonic tissue miRNAs in different colonic cell populations [both epithelial and immune subsets] during active and quiescent UC as well as in HC [Figure 1E]. Briefly, downstream genes of the IL-4 and IL-13 pathway, including cytokine receptors [in particular *IL13RA1* and *IL4R*] and signal regulators [*JAK1*, *SOCS1*, *STAT3*, and *STAT6*] were detectable in a larger proportion of cells with potentially altered expression in active UC compared to HC.

Collectively, differentially expressed colonic tissue miRNAs [including miR-223-3p, miR-20a-5p, miR-155-5p, miR-146a-5p, and miR-1-3p] showed potential involvement in regulation of the IL-4 and IL-13 signalling pathway in both active and quiescent UC, dysregulation of which was further supported by the pathway-specific gene expression analysis.

### 3.2. Sequencing of FACS-enriched colonic epithelial cells shows cell type-specific miRNA expression during colonic inflammation in UC

The role of IL-4 and IL-13 in mediating permeability of the epithelial barrier, as well as their relationship to the pathogenesis of UC, has been previously described.^29^ Additionally, colonic biopsies largely consist of epithelial cells, with comparatively fewer cells of other lineages.^25^ Therefore, as we have shown that the key player* IL-13* was overexpressed during active and quiescent UC in the colonic tissue, and different epithelial cell populations [such as differentiated and undifferentiated colonocytes, as well as goblet and enteroendocrine cells] expressed downstream genes of this signalling pathway, we further explored colonic epithelial cells during UC more deeply by analysing miRNA dysregulation in two distinct cell populations—crypt-top [CD66a+] and crypt-bottom [CD44+] colonic epithelial cells.

**Figure 2.**
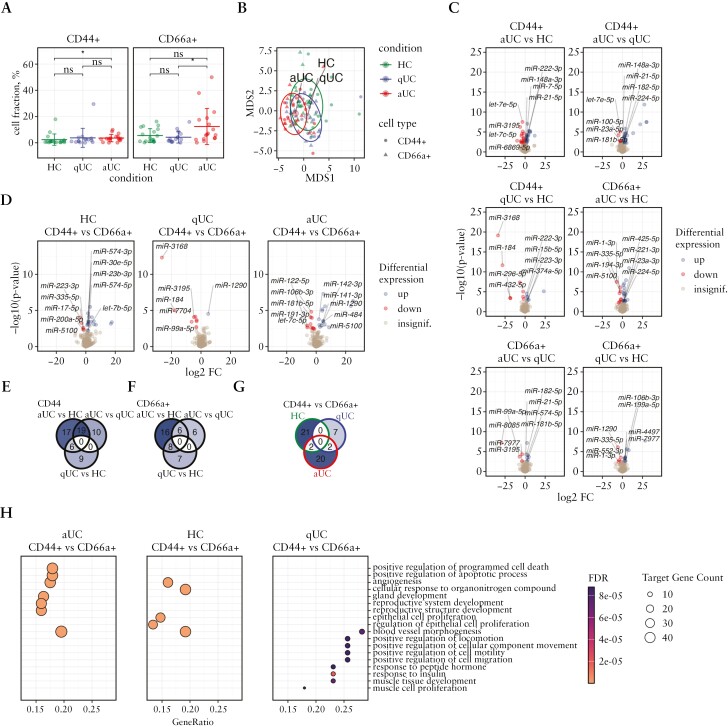
Sequencing data reveal differentially expressed miRNAs in colonic epithelial cells of patients with active and quiescent UC. [A] FACS of colonic epithelial cells and distribution of crypt-top CD44^−^CD66a^+^ and crypt-bottom CD44^+^CD66a^−^ epithelial cell types in inflammatory [aUC] [*n* = 16] and non-inflammatory [qUC and HC] [*n* = 15 and 17] colon tissues. Each dot represents a sample from the patients of the second study group. Mean ± SD of each group is represented by vertical lines. To compare the groups a non-parametric Mann–Whitney U test was performed, *p < 0.05. [B] MDS plot showing the similarity structure of the miRNA transcriptomes in crypt-top [CD66a^+^] and crypt-bottom [CD44^+^] colonic epithelial cell populations in active UC [aUC] [*n* = 16], quiescent UC [qUC] [*n* = 15], and HC [*n* = 17] based on normalized expression values. Dots represent samples shaped by cell population. Dot colours represent condition. The centroid of ellipses corresponds to the condition group mean; the shapes are defined by covariance within the group. [C, D] Volcano plots of differentially expressed miRNAs in crypt-top [CD66a^+^] and crypt-bottom [CD44^+^] colonic epithelial cell populations in aUC [*n* = 16], qUC [*n* = 15], and HC [*n* = 17]. Colours indicate significantly [FDR < 0.05] differentially expressed miRNAs with an absolute value of log_2_FC > 1 between compared groups. [E–G] Venn diagrams representing the numbers of commonly and uniquely differentially expressed miRNAs in [E] crypt-bottom [CD44^+^] and [F] crypt-top [CD66a^+^] epithelial cell populations in different UC activity, and [G] between crypt-bottom and crypt-top cells in the same condition. [H] Overrepresented pathways with the top five FDR values between crypt-top [CD66a^+^] and crypt-bottom [CD44^+^] colonic epithelial cell populations during aUC [*n* = 16], qUC [*n* = 15], and HC [*n* = 17] identified by miRNA–target gene set enrichment analysis. Dot size represents the number of miRNA gene–target counts in the significantly enriched [FDR < 0.05] GO categories.

FACS was applied to select and enrich for crypt-bottom and crypt-top colonic epithelial cells from active and quiescent UC patients and healthy controls using CD44^+^ and CD66a^+^ surface markers [Supplementary Figures S2 and S3]. Interestingly, analysis of flow cytometry showed a significant [FDR < 0.05] increase in crypt-bottom [CD44^+^] cells in active UC compared to HC [Figure 2A], suggesting a potential inflammation-stimulated cell proliferation.^30,31^

Using sequencing, 436 unique miRNAs were found to be expressed in crypt-bottom [CD44^+^] and crypt-top [CD66a^+^] epithelial cells. Although the miRNA transcriptomes of these colonic epithelial cell populations were rather similar [Figure 2B], significant changes in expression profiles were observed within and between cell populations in different stages of the disease [Figure 2C and D; Supplementary Table S3]. Initially, pairwise comparisons were performed in the same epithelial cell population to identify UC inflammation-associated miRNAs. As in the tissue data, the number of differentially expressed miRNAs [FDR < 0.05 and |log_2_FC| > 1] in both colonic epithelial cell populations were gradually increased depending on disease activity [Figure 2C]. Among deregulated molecules, no miRNAs were commonly differentially expressed across all three comparisons [active UC vs HC; quiescent UC vs HC; active UC vs quiescent UC] in both crypt-bottom [CD44^+^] and crypt-top [CD66a^+^] colonic epithelial cells. However, six miRNAs [miR-15b-5p, miR-222-3p, miR-223-3p, miR-194-3p, miR-3195, and miR-574-3p] were identified as commonly differentially expressed in crypt-bottom [CD44^+^] cells and eight miRNAs [let-7c-5p, miR-106b-3p, miR-125b-5p, miR-1-3p, miR-1290, miR-194-3p, miR-335-5p, and miR-552-3p] in crypt-top [CD66a^+^] cells in active and quiescent UC when compared to HC [Figure 2E and F]. Similar to the colonic tissue data, the majority of the overrepresented pathways in both colonic epithelial cell populations at both stages of disease activity overlapped and included signalling pathways such as ‘Signaling by Interleukins’ [R-HSA-449147], ‘Interleukin-4 and Interleukin-13 signaling’ [R-HSA-6785807], and ‘Signaling by Receptor Tyrosine Kinases’ [R-HSA-9006934] [Supplementary Figure S5 and Table S4]. This supports that observations in the colon biopsy samples were mainly driven by colonic epithelial cells.

Further, the response of distinct epithelial cell populations to inflammation was determined by performing pairwise comparisons with the separate populations of colonic epithelial cells. Interestingly, 24 miRNAs were identified to be differentially expressed in active UC, nine miRNAs in quiescent UC, and 22 miRNAs in HC when compared crypt-bottom [CD44^+^] and crypt-top [CD66a^+^] colonic epithelial cells [Figure 2D; Supplementary Table S3]. Notably, the vast majority of identified differentially expressed miRNAs between distinct epithelial cell types in different stages of inflammation were found to be uniquely dysregulated. Only two commonly differentially expressed miRNAs were observed in each of the two comparison groups: miR-106b-3p and miR-1290 in active UC CD44^+^ vs CD66a^+^ and quiescent UC CD44^+^ vs CD66a^+^, and miR-296-5p and miR-432-5p in active UC CD44^+^ vs CD66a^+^ and HC CD44^+^ vs CD66a^+^ [Figure 2G]. This suggests that even at different disease activity stages there are cell population-specific responses, in terms of miRNA expression. The results of GSEA [using GO terms] of deregulated miRNAs in the active UC, quiescent UC, and HC groups revealed that overrepresented processes between crypt-bottom [CD44^+^] compared to crypt-top [CD66a^+^] cells were mainly related to cell differentiation and motility in both active UC and HC [Figure 2H], suggesting that in inflamed and healthy colon mucosa these pathways are differentially regulated between the cell types. Additionally, significant enrichment in ‘epithelium migration’ [GO:0090132] and ‘epithelial cell migration’ [GO:0010631] between crypt-bottom [CD44^+^] and crypt-top [CD66a^+^] cells was uniquely identified only in active UC among the most overrepresented biological processes. Target genes of differentially expressed miRNAs between cell populations in the quiescent UC group were mainly related to cell migration and were least different between those populations.

In summary, despite the significant overlap of aberrantly expressed miRNAs in both colonic epithelial cell populations within regulatory signalling pathways, GSEA results revealed unique involvement of differentially expressed miRNAs between cell populations in processes related to intestinal barrier integrity.

### 3.3. miRNAs in crypt-top [CD66a^+^] and crypt-bottom [CD44^+^] colonic epithelial cells exhibit a co-expression pattern which is related to UC activity

First, to reveal the relationship between individual miRNA expression levels and endoscopic Mayo subscore in crypt-top [CD66a^+^] and crypt-bottom [CD44^+^] colonic epithelial cells, Spearman correlation analysis was used. In crypt-bottom [CD44^+^] and crypt-top [CD66a^+^] colonic epithelial cells, a number [*n* = 34 and *n* = 23, respectively] of moderate positive [0.4 < rho < 0.7; FDR < 0.05] and a few [*n* = 6 and *n* = 7, respectively] moderate negative [−0.7 < rho < −0.4; FDR < 0.05] correlations were observed among the normalized miRNA expression levels and endoscopic Mayo subscore [Supplementary Figure S6; Supplementary Tables S5 and S6]. Notably, analysis not only resulted in a substantial overlap in disease activity-associated miRNAs between both colonic epithelial cell populations [29 common moderately correlating miRNAs], but also revealed a few cell population-unique correlations.

**Figure 3.**
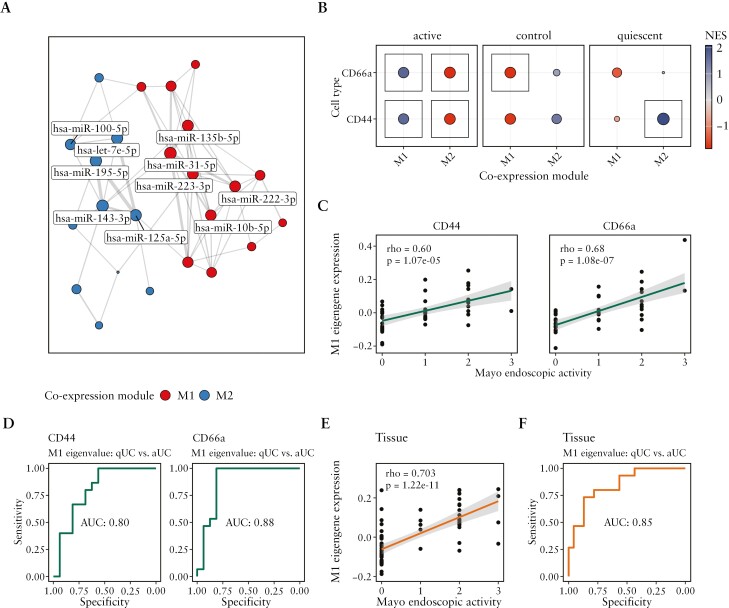
WGCNA in colonic epithelial cell populations reveals miRNA co-expression modules which reflect endoscopic activity of UC. [A] Network displaying identified co-expression modules [M1 and M2] in crypt-top [CD66a] and crypt-bottom [CD44] colonic epithelial cells of patients with active [aUC] [*n* = 16], quiescent UC [qUC] [*n* = 15], and control individuals [*n* = 17]. The colour and size of the node represents distinct modules and strength of connectivity, respectively. [B] Dot plot showing normalized enrichment score [NES] of modules M1 and M2 in crypt-top [CD66a] and crypt-bottom [CD44] colonic epithelial cells of patients with active UC, quiescent UC, and control individuals. The colour and size of the dot represents the value and absolute value of NES, respectively. The box marks significant value [*p*_adj,_ < 0.05]. Plots [C] and [E] show the correlation between module M1 eigengene value and Mayo endoscopic activity in crypt-bottom [CD44] and crypt-top [CD66a] colonic epithelial cells as well as colon tissue, respectively. rho—Spearman correlation coefficient. Each dot represents a different sample. Plots [D] and [F] show the AUC-ROC curves reflecting the performance of M1 module eigengene value at distinguishing between active UC [aUC] and quiescent UC [qUC] in crypt-bottom [CD44] and crypt-top [CD66a] colonic epithelial cells as well as in colon tissue, respectively.

Subsequently, we performed more complex analysis and evaluated if certain colonic epithelial cell miRNAs of both populations are co-expressed together. First, WGCNA performed on all miRNAs of colonic epithelial cell populations uncovered the miRNA co-expression network [Figure 3A] and identified two co-expression modules [M1 and M2]. Module M1 comprised 13 miRNAs [miR-10b-5p, miR-182-5p, miR-146a-5p, miR-196b-5p, miR-222-3p, miR-27a-3p, miR-221-3p, miR-194-3p, miR-183-5p, miR-223-3p, miR-574-5p, miR-135b-5p, and miR-31-5p], while module M2 consisted of 11 miRNAs [let-7b-5p, miR-143-3p, miR-125a-5p, miR-15b-5p, let-7e-5p, miR-5100, miR-181b-5p, miR-1-3p, miR-125b-5p, miR-100-5p, and miR-195-5p]. Notably, certain miRNAs in module M1 [e.g. miR-31-5p, miR-223-3p, and miR-10b-5p] were also previously identified as important regulators of genes involved in interleukin signalling pathways [Figure 2H]. Subsequent module enrichment analysis based on the evaluation of normalized enrichment score [NES] [Figure 3B] further revealed that module M1 was significantly enriched in both crypt-top [CD66a^+^] and crypt-bottom [CD44^+^] epithelial cells of patients with active UC (NES = 1.71 [*p*_adj._ = 9.7 × 10^−3^] and 1.67 [*p*_adj._ = 2.9 × 10^−2^], respectively), while in both cell populations of control group individuals it was decreased [NES = −1.79 [*p*_adj._ = 7.7 × 10^−3^] and −1.74 [*p*_adj._ = 5.0 × 10^−2^], respectively). Interestingly, the enrichment values for module M2 were opposite to that observed for M1, meaning the normalized expression of module M2 in both crypt-top [CD66a^+^] and crypt-bottom [CD44^+^] epithelial cell populations of patients with active UC was significantly decreased (NES = −1.84 [*p*_adj._ = 9.7 × 10^−3^] and −1.80 [*p*_adj._ = 2.9 × 10^−2^], respectively), while it was significantly enriched exclusively in crypt-bottom [CD44^+^] cells of patients with quiescent UC (NES = 2.08 [*p*_adj._  = 3.3 × 10^−4^]).

Further, we focused on the potentially pro-inflammatory module M1 and explored whether the expression of this module in each of the studied epithelial cell populations is associated with clinical characteristics of UC. Therefore, Spearman correlation analysis was used to check for an association between endoscopic Mayo subscore and module M1 eigengene value [summarized module expression value]. Analysis showed a significant moderate positive correlation in both crypt-top [CD66a^+^] and crypt-bottom [CD44^+^] cells (rho = 0.68 [*p* = 1.08 × 10^−7^] and 0.60 [*p* = 1.07 × 10^−5^], respectively) [Figure 3C]. Next, AUC-ROC analysis was used to assess the performance of the module M1 eigengene value in distinguishing between active and quiescent UC in each of the studied colonic epithelial cell populations. In crypt-bottom [CD44^+^] cells, analysis gave an AUC value of 80.0% (confidence interval [CI]: 63.6–96.4%], while in the crypt-top [CD66a^+^] cells M1 eigengene expression value demonstrated even better performance with an AUC value of 87.9% [CI: 74.0–100.0%] when separating quiescent vs active UC [Figure 3D]. To evaluate the concordance of the epithelial cell population-derived data with the situation in colon tissue, we calculated module M1 eigengene values in the tissue and performed analogous analysis as in the crypt-top and crypt-bottom cell populations. In colon tissue, the module M1 eigenvalue showed strong a positive correlation with endoscopic Mayo subscore (rho = 0.703 [*p* = 1.22 × 10^−11^]) [Figure 3E] with an AUC of 85.0% [CI: 72.2–97.1%] [Figure 3F]. These results indicated high resemblance between whole tissue and colonic crypt-top [CD66a^+^] epithelial cells.

Generally, the results uncovered potential UC endoscopic activity-related miRNA co-expression patterns that are not only characteristic for both crypt-top and crypt-bottom colonic epithelial cell populations, but also reflect the overall situation in the more heterogeneous colon tissue during UC.

## 4. Discussion

Although UC is a well-studied complex disease and huge efforts have been made to explore its molecular mechanisms, the disease pathogenesis remains largely unclear.^32^ In particluar, there is a substantial knowledge gap regarding expression patterns of regulatory non-coding miRNA in UC in a cell type-specific context. Thus, here we provide an overview of the miRNA expression in unsorted whole colonic mucosa samples of UC patients, present detailed colonic epithelial cell population-specific miRNA expression profiles from active and quiescent UC patients, and describe differences in miRNA expression patterns between two distinct—crypt-bottom [CD44^+^] and crypt-top [CD66a^+^]—cell populations. Furthermore, we describe putative biological pathways in which deregulated miRNAs of UC colonic epithelial cell populations might be involved, identify a potential inflammatory miRNA co-expression module, determine its associations with endoscopic disease activity, and evaluate its performance in distinguishing between active and quiescent stages of UC.

Most importantly, we determined distinct responses in miRNA expression of different colonic epithelial cell populations during UC. Our findings showed that in colon crypt-bottom [CD44^+^] cells (compared to crypt-top [CD66a^+^] cells) inflammation promoted/suppressed the expression of several miRNAs possibly involved in cell proliferation, differentiation, and/or permeability of the intestinal barrier. For example, let-7c-5p showed considerable down-regulation and miR-501-3p up-regulation in crypt-bottom [CD44^+^] cells during active UC. It has previously been shown that overexpression of let-7c-5p as well as inhibition of miR-501-3p can reduce the proliferation of colorectal cancer cells.^33,34^ Thus, deregulation of these miRNAs might be related to the relative increase of crypt-bottom [CD44^+^] cells in active UC when compared to controls, as has been shown in our flow cytometry experiment. On the other hand, we observed increased expression of miR-1-3p and decreased expression of miR-125b-5p in crypt-bottom compared to crypt-top cells only during active UC. Both miRNAs were shown to be involved in barrier function dysregulation, where a decrease of miR-125b-5p^35^ and increase of miR-1-3p^36^ contribute to disruption of the epithelial barrier in colon tissue. This would suggest that the epithelial barrier is already impaired in crypt-bottom epithelial cells during active UC; however, it remains unclear if this is a UC-specific event or rather a normal cell response to inflammation in the gut.

Furthermore, our analysis showed that not only are certain individual miRNAs associated with the endoscopic Mayo subscore, but they also form an inflammation-related co-expression network, which directly correlates with clinical disease activity. Of two identified miRNA co-expression modules in both crypt-bottom [CD44^+^] and/or crypt-top [CD66a^+^] cells, module M1 was significantly enriched in both epithelial cell populations of active UC patients and comprised miRNAs such as miR-31-5p, miR-135b-5p, miR-27a-3p, miR-222-3p, and miR-223-3p. In the literature, these small non-coding RNAs are also reported to be up-regulated in inflamed, non-inflamed and/or pre-cancerous colon tissues as well as in faeces [specifically, miR-223-3p] of UC patients^37–42^ and are involved in the regulation of inflammatory response [e.g. miR-222-3p targets *SOCS1* and activates STAT3 signalling].^43–45^ Additionally, we observed that all miRNAs belonging to module M1 were involved in Reactome pathways related to interleukin signalling by targeting various validated genes [e.g. *FOXO3*, *IGF1R*, *ICAM1*, *STAT6*, *STAT1*, *STAT5A*, and *CCND1*]. Interestingly, when comparing crypt-top and crypt-bottom cell population-derived module M1 performance and association results to colon tissue, both the correlation coefficient and AUC values in tissue were more like those observed in crypt-top [CD66a^+^] cells. This may be at least partially explained by the cellular composition of the colon mucosa, the most abundant cell type in the mucosal layer being absorptive colonocytes.^46^ By contrast, the second identified miRNA co-expression module M2 showed an inverse correlation with the endoscopic Mayo subscore and was exclusively enriched in crypt-bottom [CD44^+^] colonic epithelial cells of quiescent UC patients, suggesting its anti-inflammatory properties. For example, among module M2 miRNAs we identified let-7e-5p, which, together with other let-7 miRNAs, has been previously shown to affect maintenance of cell differentiation.^47^ Additionally, in the intestinal epithelium let-7 family member let-7b appears to be among the highest-expressed miRNAs in the let-7 group/family [Supplementary Figure S6]. Together, these observations suggest the relevance of let-7 miRNAs during intestinal inflammation via maintenance of stemness of crypt-bottom [CD44^+^] cells and thereby explain their expression correlation with disease activity, exclusively in undifferentiated colonic epithelial cells.

Finally, we described potential involvement of differentially expressed miRNAs in regulatory biological pathways. Our initial small RNA-seq of colonic mucosa biopsies from active and quiescent UC compared to healthy controls, at first, revealed multiple deregulated miRNAs, which were significantly enriched in inflammation- and intestinal epithelial barrier function-related biological pathways. Interestingly, miRNAs enriched in interleukin biological pathways, such as IL-4 and IL-13 signalling, were deregulated not only in active but also in quiescent UC, suggesting lasting derangement of this pathway in UC mucosa. The IL-4 and IL-13 pathway is known to differentially regulate epithelial chloride secretion and cause epithelial barrier dysfunction.^48^ It has been shown that large amounts of IL-13 are produced in colon mucosa of UC patients and thereby impair epithelial barrier function by affecting epithelial apoptosis, tight junctions, and restitution velocity.^29,49^ We also confirmed the increased expression of the IL-13 gene during the course of UC when analysing active and quiescent UC patient colon tissue samples. By contrast, some studies report decreased mucosal amounts of IL-13 in active UC.^50^ Nevertheless, attempts are still being made to adapt the inhibition of IL-13-based treatment to induce UC remission (e.g. clinical trials of anti-IL-13 monoclonal antibodies [tralokinumab and anrukinzumab]^51^ and preclinical studies of anti-IL-Rα2^52^). Similar to the results in colonic biopsies, both crypt-bottom [CD44^+^] and crypt-top [CD66a^+^] epithelial cell populations showed deregulation in miRNAs during UC, the targets of which were significantly enriched in the IL-4 and IL-13 signalling pathway. Since IL-4 and IL-13 cytokines are predominantly produced by immune cells,^53^ the expected regulatory action of deregulated miRNAs in colonic epithelial cells would be downstream targets of the pathway, such as *STAT3*, *FOXO3*, and *SOCS1*. During active UC in both crypt-bottom [CD44^+^] and crypt-top [CD66a^+^] cells, we found miR-221-3p, miR-182-6p, miR-222-3p, and miR-31-5p to be up-regulated, which are known to target the *FOXO3* gene.^22^ Up-regulation of the aforementioned miRNAs, theoretically, would lead to decreased expression of the *FOXO3* gene, as already observed in colonic mucosa of UC patients.^54^ This, in turn, may lead to more severe colonic inflammation during UC.^55^ Additionally, both crypt-bottom [CD44^+^] and crypt-top [CD66a^+^] cells of patients with active UC had increased expression of hsa-miR-221-3p and hsa-miR-21-5p, which target the *SOCS1* gene.^22^ SOCS1 is an important regulator of IL-4 signalling, and its forced expression was shown to inhibit IL-13 signalling in epithelial cells.^56^ In addition to involvement in interleukin signalling pathways, we observed a few aberrantly expressed miRNAs between crypt-bottom [CD44^+^] and crypt-top [CD66a^+^] cells in active UC that possibly exert their biological function through regulation of epithelial cell migration, which is known to occur along the crypt–villus axis^57^ and is increased during inflammatory bowel disease.^58^ Noteworthy, the GSEA results should be treated with caution, since the selection of miRNA targets significantly affects the results.^59^ However, currently, there are no methods to solve this issue, since miRNA target prediction as well as its dosage to affect target expression remain unsolved problems in the field.

In summary, our study determined crypt-bottom [CD44^+^] and crypt-top [CD66a^+^] colonic epithelial cell-specific miRNA deregulation in UC in a cell type- and disease stage-dependent manner. We also revealed cell population-specific miRNA expression patterns and networks as well as their associations with clinical disease activity. Furthermore, we unveiled the potential functional role of differentially expressed miRNAs and observed their possible involvement in biological pathways associated with maintenance of intestinal barrier function in active as well as quiescent UC, in both epithelial cell populations. Together, these observations not only highlight the regulatory importance of miRNAs in distinct colonic epithelial cell populations during the pathogenesis of UC, but also provide potential miRNA candidates for the development of new treatment strategies to maintain the remission of mucosal inflammation.

## Supplementary Material

jjae108_suppl_Supplementary_Figures_S1-S7

## Data Availability

The small RNA-seq data underlying this article are available in the Gene Expression Omnibus [GEO] Database and can be accessed with accession numbers GSE185101 and GSE185102. Supplementary Tables S1–S6 are available upon request from the corresponding author.

## Conference Presentation

Part of this work was presented at the 16th Congress of ECCO Virtual—Inflammatory Bowel Diseases 2021, July 2–3 & 8–10, 2021.
