# Supplementary material for: The microRNA Expression in Crypt-Top and Crypt-Bottom Colonic Epithelial Cell Populations Demonstrates Cell-Type Specificity and Correlates with Endoscopic Activity in Ulcerative Colitis
Source: J Crohns Colitis. 2024 Jul 18;18(12):2033–44. doi: 10.1093/ecco-jcc/jjae108 (PMC11637558; doi:10.1093/ecco-jcc/jjae108)
Supplement: jjae108_suppl_Supplementary_Figures_S1-S7 [file jjae108_suppl_supplementary_figures_s1-s7.pdf]

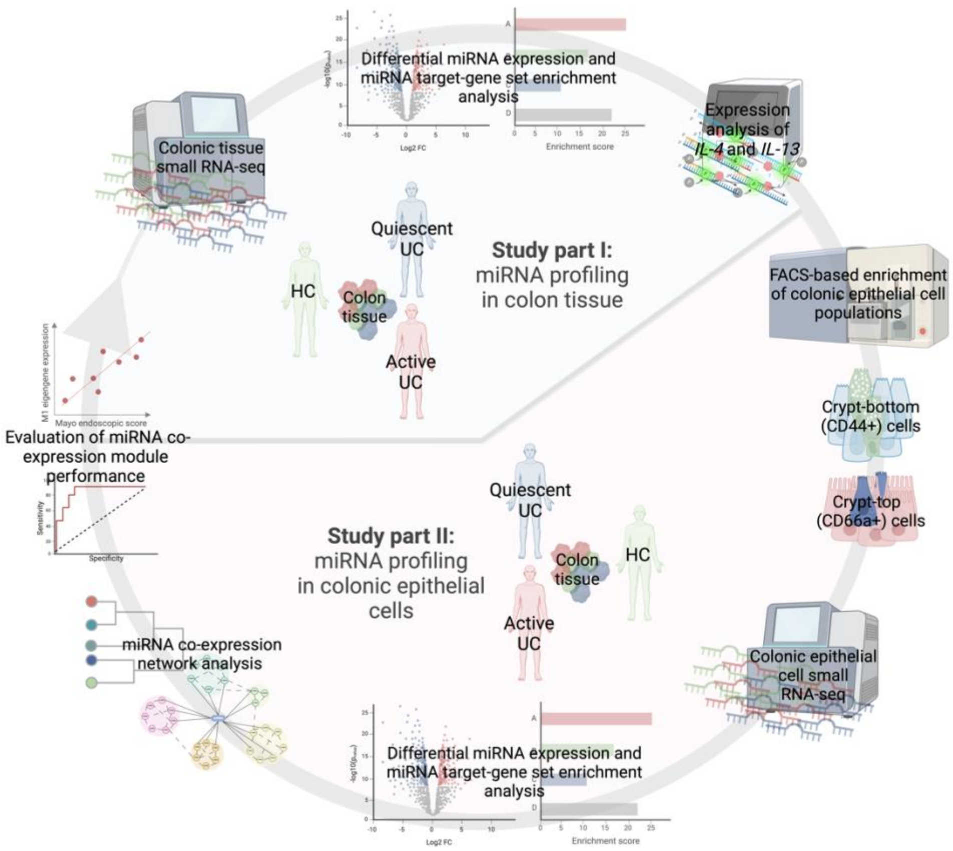

Supplementary Figure S1: Study design flowchart. aUC – active UC, qUC – quiescent UC, HC – healthy control. Created with BioRender.com

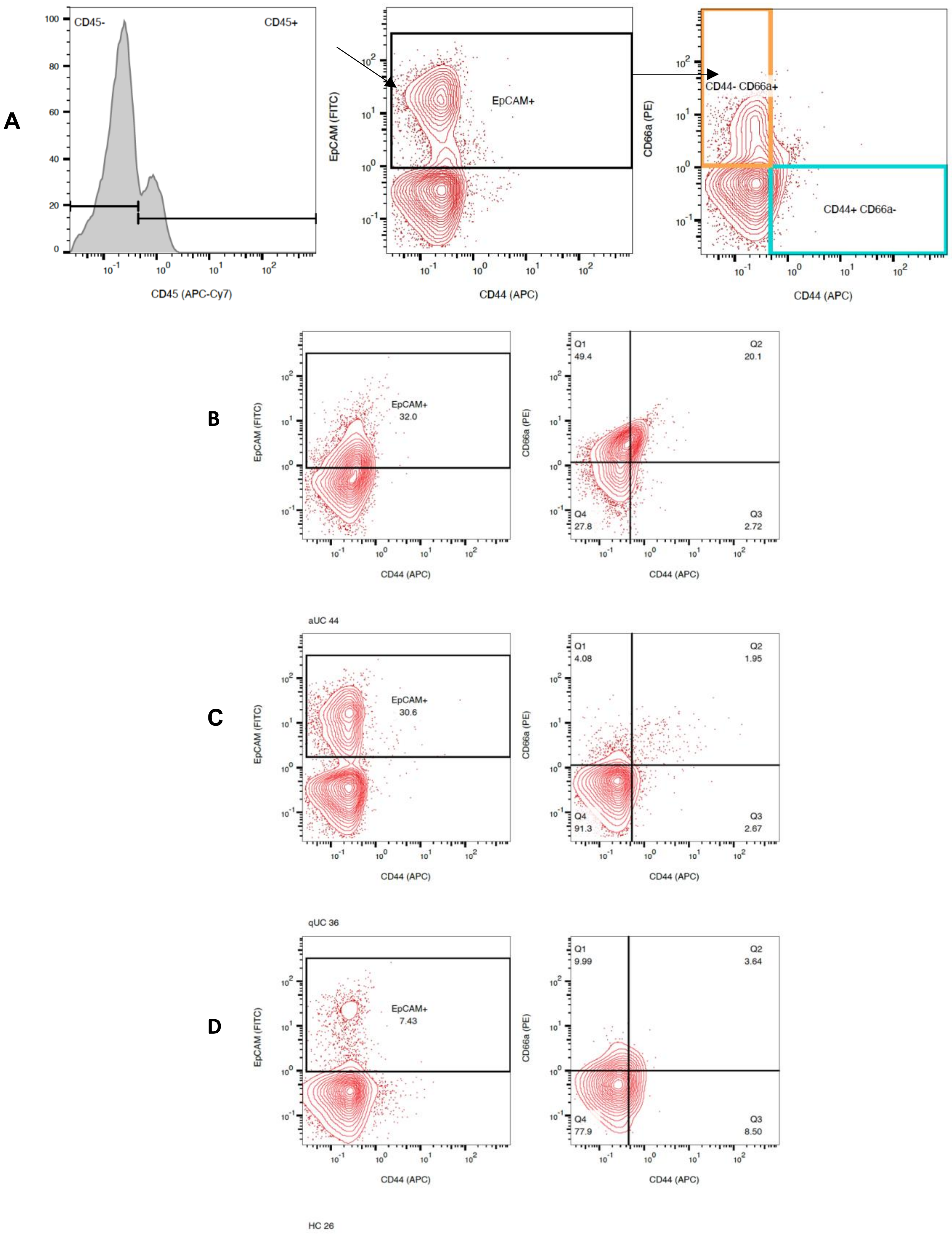

Supplementary Figure S2: Fluorescence-activated cell sorting (FACS) of crypt-bottom (CD44^{+}) and crypt-top (CD66a^{+}) colonic epithelial cells from patients with active and quiescent UC and healthy controls (HC). (A) Gating strategy for FACS isolation of crypt-top CD44+/CD66a- and crypt-bottom CD44-/CD66a+ epithelial cell populations: selection of epithelial cells by CD45 exclusion, EpCAM inclusion and further isolation of populations by CD44 and CD66a expression. **(B-D)** Representative plots of flow cytometry data showing distribution of CD44+/CD66a- and CD44-/CD66a+ colonic epithelial cell populations during **(B)** active UC (aUC), **(C)** quiescent UC (qUC), and **(D)** in healthy controls (HC).

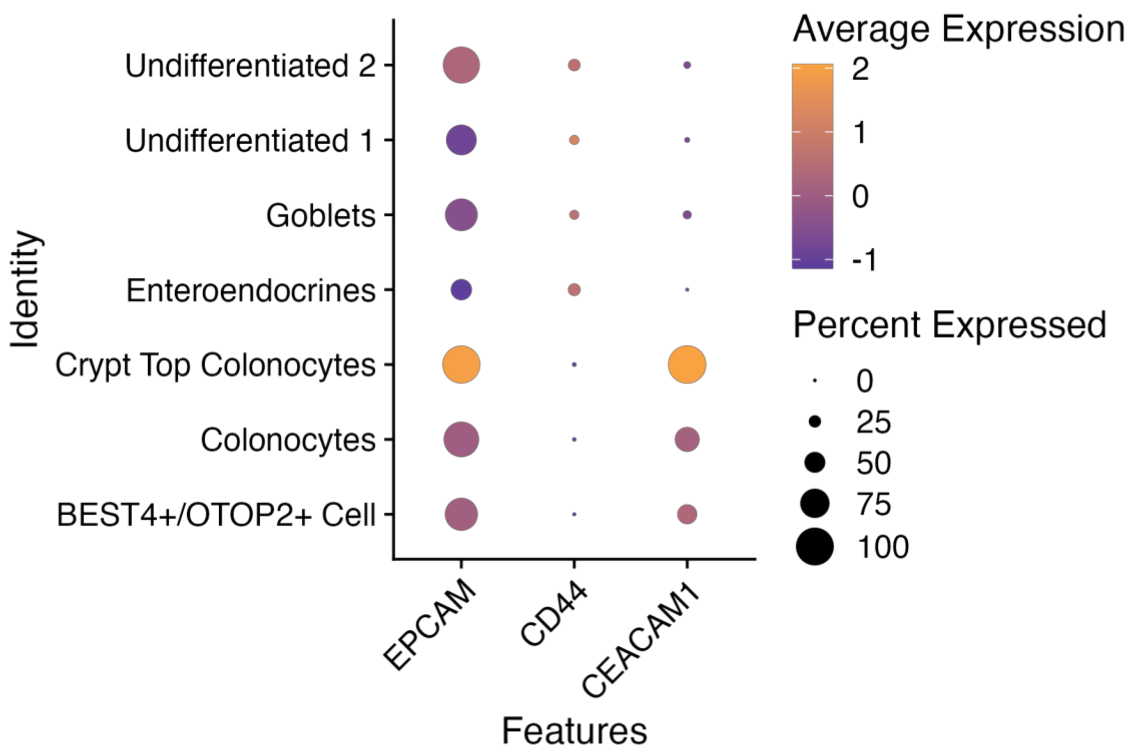

Supplementary Figure S3: The dot plot representing expression of the main used cell surface markers for FACS of colonic epithelial crypt-top (EpCAM+/CD44-/CD66a+) and crypt-bottom (EpCAM+/CD44+/CD66a) cell populations. *EPCAM* is expressed in all colonic epithelial cells, *CD44* is a marker for crypt-bottom cells (undifferentiated, Goblet and enteroendocrine cells), *CEACAM1* (coding CD66a+) is a marker for crypt-top cells (crypt top colonocytes, colonocytes and BEST4+/OTOP2+ cells). The average expression of genes and percentage of cells expressing the genes are represented by dot color and size, respectively. The single-cell RNA-seq data used for preparation of this plot was downloaded from GEO database (accession number GSE116222).

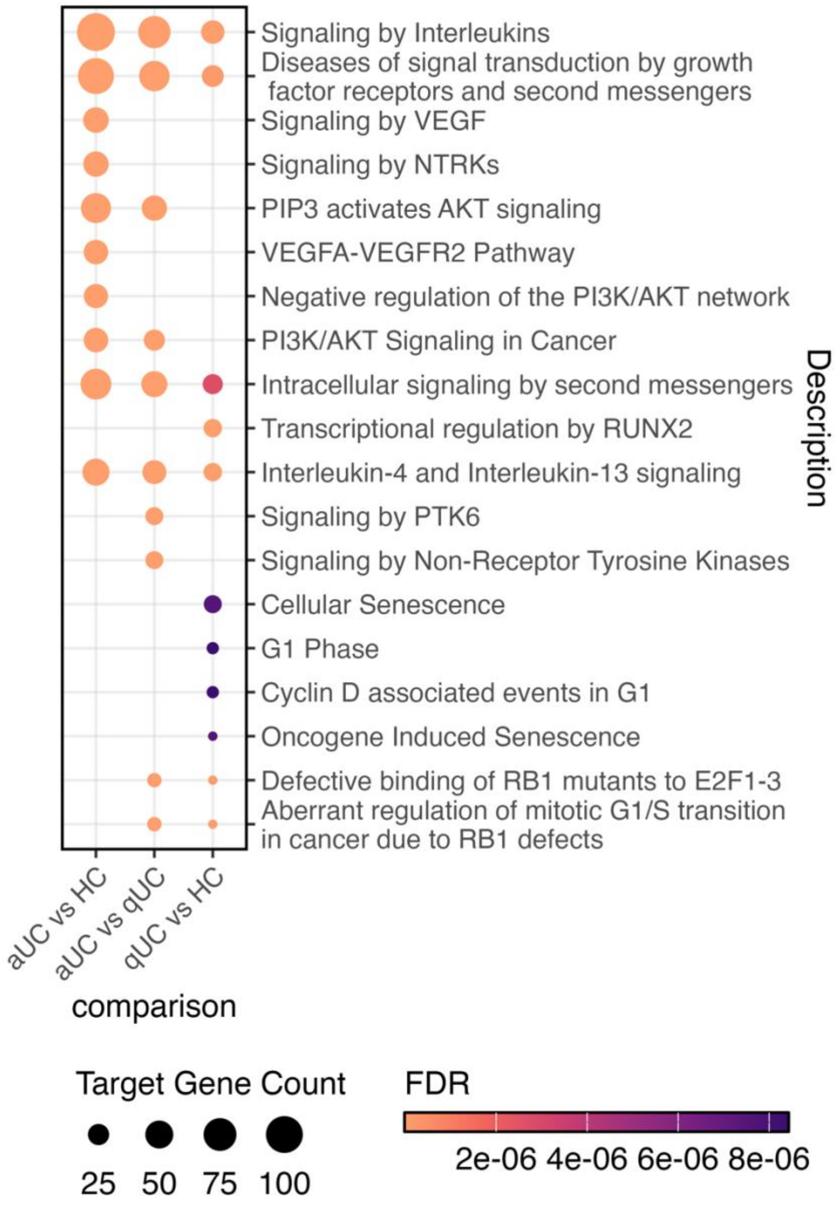

Supplementary Figure S4: Small RNA-seq defines differentially expressed miRNAs involved in inflammation-associated pathways in active and quiescent UC tissues. Dot plot shows top 10 overrepresented pathways in active (aUC) (n=23) and quiescent UC (qUC) (n=20) tissues identified by miRNA set enrichment analysis Dot size represents the number of miRNA-target gene count in the significantly enriched (FDR < 0.05) Reactome pathways.

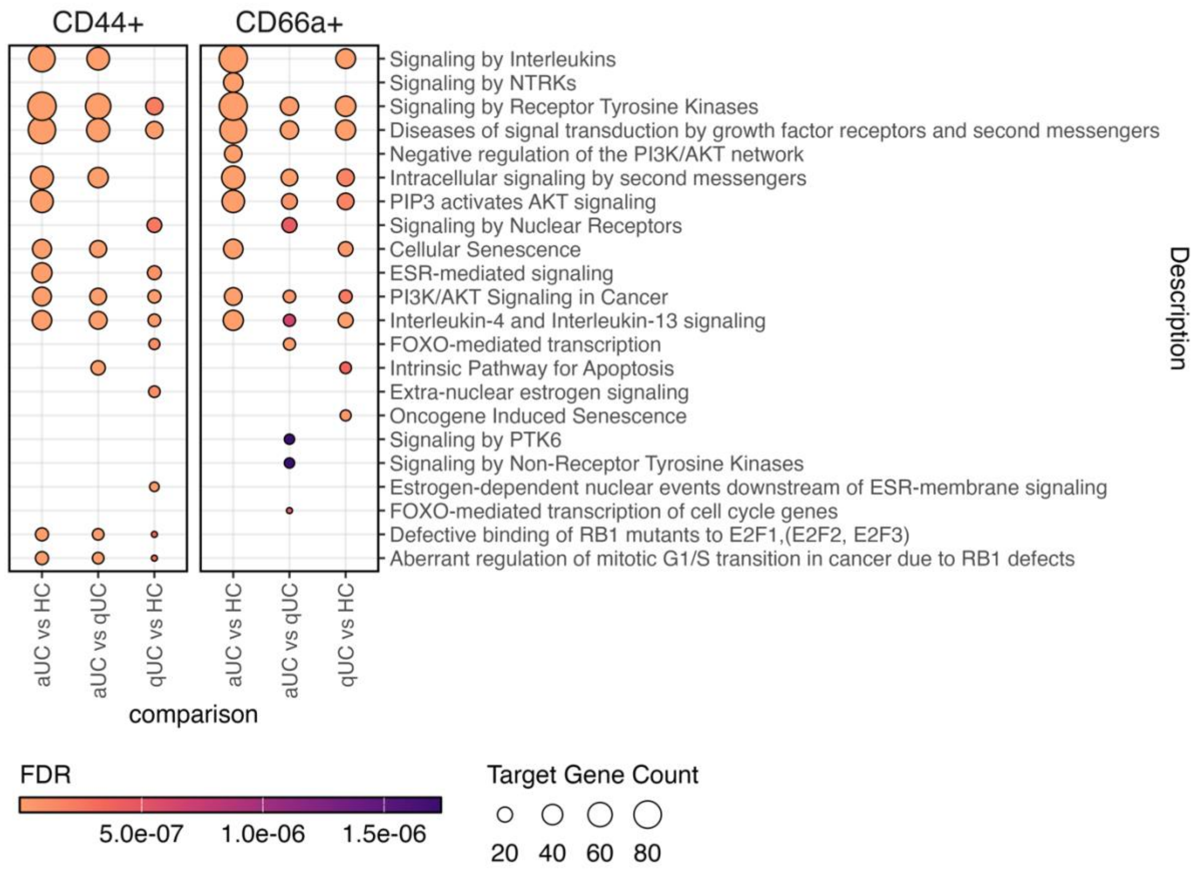

Supplementary Figure S5: Aberrantly expressed miRNAs of crypt-top (CD66a+) and crypt-bottom (CD44+) colonic epithelial cells are involved in UC-related processes. Top 10 overrepresented pathways within crypt-top (CD66a+) and crypt-bottom (CD44+) colonic epithelial cell populations during active (aUC) (n=16), quiescent UC (qUC) (n=15) and in controls (HC) (n=17) identified by miRNA-target gene set enrichment analysis. Dot size represents the number of miRNA gene-target count in the significantly enriched (FDR < 0.05) Reactome pathways.

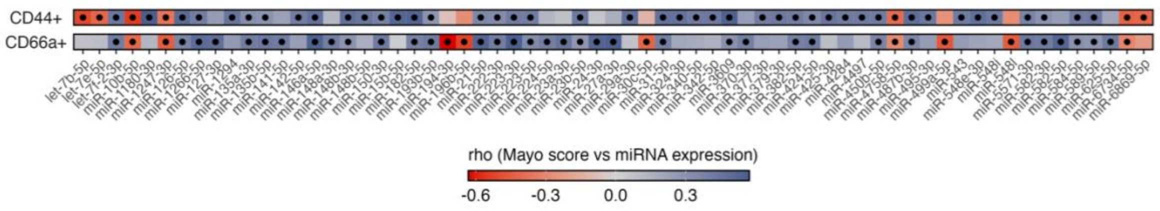

Supplementary Figure S6: A heatmap showing correlations between miRNA expression and endoscopic Mayo subscore in crypt-bottom (CD44+) (n=48) and crypt-top (CD66a+) (n=48) colonic epithelial cell populations. Colour of the box represent the value of Spearman's correlation coefficient (rho). Dots mark significant correlations (FDR < 0.05) in each cell population.

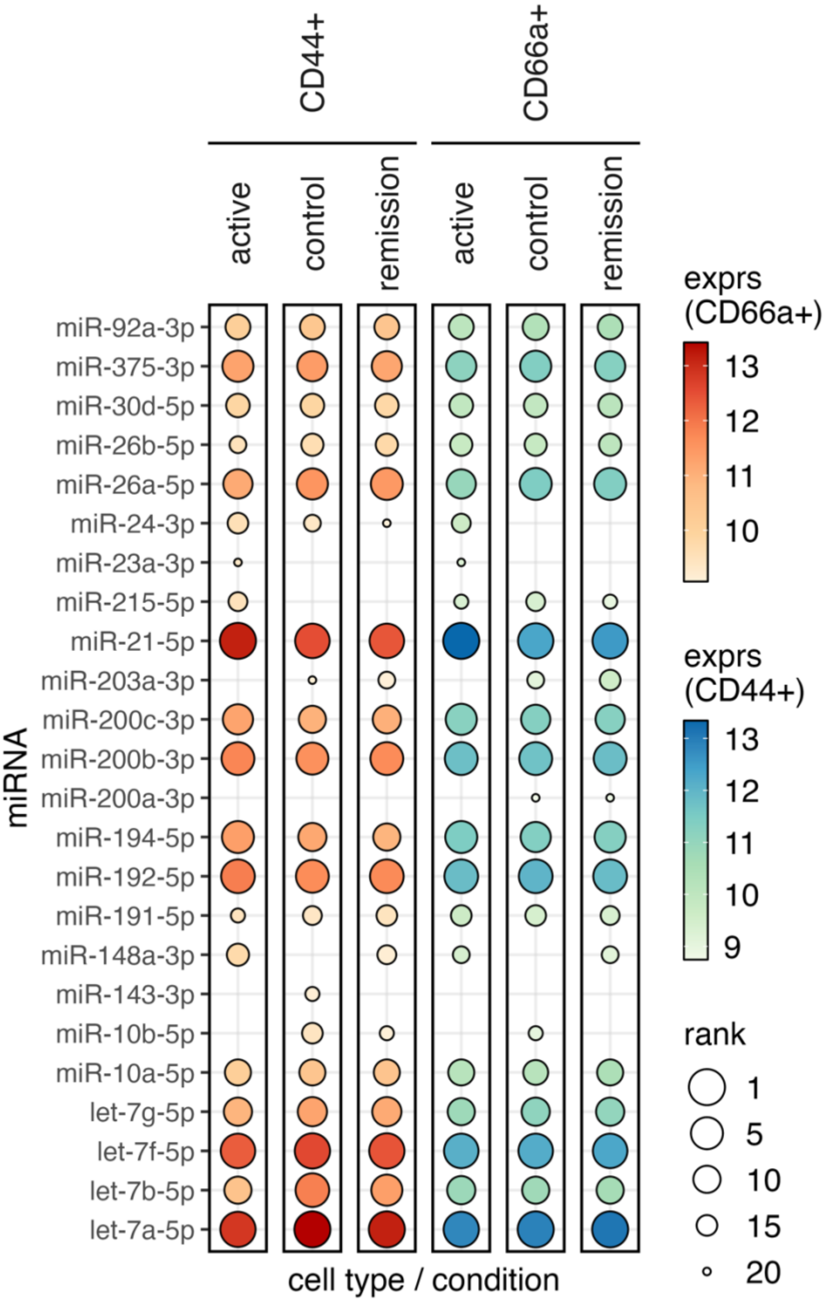

Supplementary Figure S7: The most abundant (top 20) miRNA expression in colonic epithelial cell CD44+ and CD66a+ populations from UC patients (active and remission) and healthy controls. In each cell population and condition, the median normalized expression levels are represented by color, and rank by dot size. Most abundant miRNAs are ranked in descending order, to be precise, the highest expression value is ranked as 1.

49 Spadina Ave. Suite 200
Toronto ON M5V 2J1 Canada
www.biorender.com

Confirmation of Publication and Licensing Rights

May 30th, 2024
Science Suite Inc.

Subscription:	*Individual*
Agreement number:	*BE26VR6SG1*
Journal name:	*Journal of Crohn's and Colitis*

To whom this may concern,

This document is to confirm that Rūta Inčiūraitė has been granted a license to use the BioRender content, including icons, templates and other original artwork, appearing in the attached completed graphic pursuant to BioRender's Academic License Terms. This license permits BioRender content to be sublicensed for use in journal publications.

All rights and ownership of BioRender content are reserved by BioRender. All completed graphics must be accompanied by the following citation: "Created with BioRender.com".

BioRender content included in the completed graphic is not licensed for any commercial uses beyond publication in a journal. For any commercial use of this figure, users may, if allowed, recreate it in BioRender under an Industry BioRender Plan.

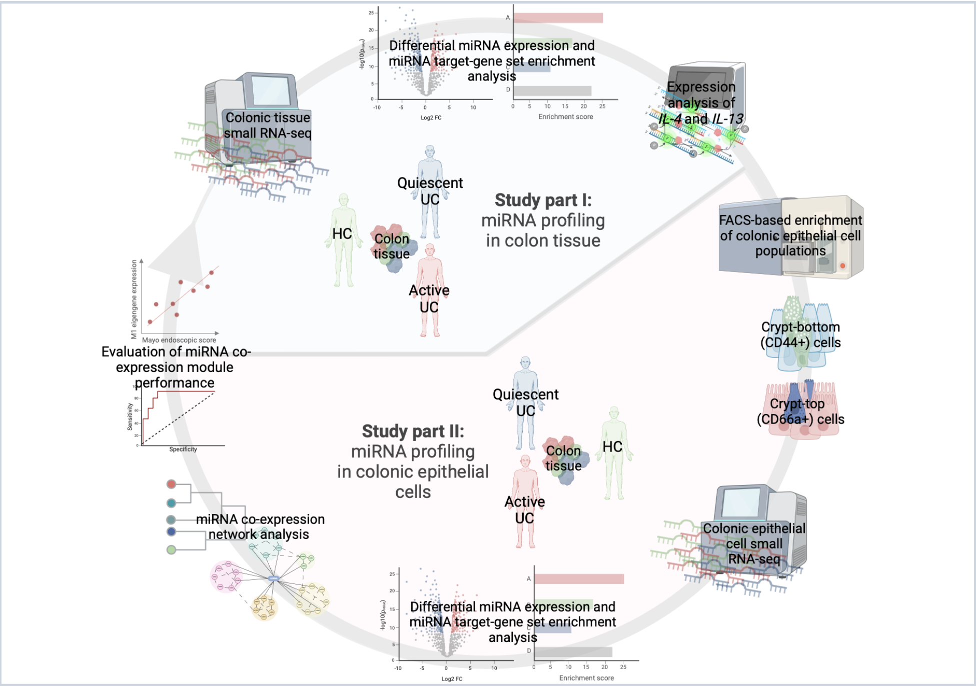

For any questions regarding this document, or other questions about publishing with BioRender refer to our BioRender Publication Guide, or contact BioRender Support at support@biorender.com.
